# Paeoniflorin Attenuates Cerebral Ischemia-Induced Injury by Regulating Ca^2+^/CaMKII/CREB Signaling Pathway

**DOI:** 10.3390/molecules22030359

**Published:** 2017-02-27

**Authors:** Yuqin Zhang, Lifei Qiao, Wen Xu, Xiaoying Wang, Huang Li, Wei Xu, Kedan Chu, Yu Lin

**Affiliations:** College of Pharmacy of Fujian University of Traditional Chinese Medicine, Fuzhou, Fujian 350122, China; zyqfj@hotmail.com (Y.Z.); qlffjtcm@sina.com (L.Q.); xwfjtcm@126.com (W.X.); wxyfjtcm@sina.com (X.W.); lihuang3413@gmail.com (H.L.); xwfjlab@163.com (W.X.)

**Keywords:** ischemic stroke, paeoniflorin, middle cerebral artery occlusion, CREB, CaMKII

## Abstract

Paeoniflorin (PF) is an active ingredient of Paeoniae Radix which possesses the neuroprotective effect. However, so far, the neuroprotective mechanism of PF has still not been fully uncovered. The Ca^2+^/Ca^2+^/calmodulin-dependent protein kinase II (CaMKII)/cAMP response element-binding (CREB) signaling pathway plays an important role in the intracellular signal transduction pathway involved in cell proliferation, cell survival, inflammation and metabolism. Herein, the neuroprotective roles of PF in the models of middle cerebral artery occlusion (MCAO) followed by reperfusion in rats and *N*-methyl-d-aspartic acid (NMDA)-induced excitotoxicity in primary hippocampal neurons were investigated. Moreover, we attempted to confirm the hypothesis that its protection effect is via the modulation of the Ca^2+^/CaMKI)/CREB signaling pathway. In this study, PF not only significantly decreased neurological deficit scores and infarct volume in vivo, but also improved neurons’ cell viability, and inhibited neurons’ apoptosis and intracellular Ca^2+^ concentration in vitro. Furthermore, PF significantly up-regulated p-CREB and p-CaMKII, and down-regulated calmodulin (CaM) in vivo and in vitro. The results indicate that the protective effect of PF on cerebral ischemia reperfusion injury is possible through regulating the Ca^2+^/CaMKII/CREB signaling pathway.

## 1. Introduction

Ischemic stroke (around 80% of the total) remains a leading cause of disability and mortality worldwide [1,2]. Due to a short therapeutic time window [3], further progression of neuronal damage, contraindications and poor efficacy or severe toxicity/side effects of agents [4,5], the remedy of ischemic stroke is still a crucial research interest.

Ischemic stroke involves the interaction of numerous pathophysiological processes, and concomitantly triggered pathogenetic mechanisms. The Ca^2+^/Ca^2+^/calmodulin-dependent protein kinase II (CaMKII)/cAMP response element-binding (CREB) signaling pathway has been shown to play a crucial role in the intracellular signal transduction pathway involved in cell proliferation, cell survival, inflammation and metabolism [6,7,8]. Accumulated evidence demonstrates a high intracellular Ca^2+^ influx in ischemic stroke. Ca^2+^ influx leads to the elevation of the Ca^2+^ concentration in neurons which disrupts the balance of ionics, then activates various Ca^2+^-dependent enzymes (such as CaMKII, nitric oxide synthase, calcineurin) and Ca^2+^-binding proteins. Ca^2+^ overload into neurons, induced by ischemic insult, may excessively activate Ca^2+^/CaM-dependent pathways and lead to irreversible cell damage [6]. CaMKII, an important member of the calcium/calmodulin-activated protein kinase family, plays a vital part in the regulation of both neuronal death and survival [9,10]. CaMKII, an important member of the calcium/calmodulin-activated protein kinase family, plays a vital part in the regulation of both neuronal death and survival [9,10]. When the Ca^2+^/calmodulin (CaM) complex combines with CaMKII, CaMKII is activated and further autophosphorylates at Thr286 to render the kinase constitutively active. Several reports have demonstrated that phosphorylation of CaMKII plays an important part in the regulation of ischemia [11,12,13]. Phosphorylation of CaMKII then activates CREB protein. CREB is a transcription factor present in numerous tissues, which plays a large regulatory role in neuronal survival, precursor proliferation, neurite outgrowth, and neuronal differentiation in certain neuronal populations [14,15,16]. Phosphorylation of CREB attenuates neuronal apoptosis by phosphorylating and subsequently activating downstream anti-apoptotic proteins [15,17].

Paeoniflorin (PF) is a monoterpene glycoside which is isolated from Radix Paeoniae (dried root of Paeonia lactiflora Pall.) and is, at the same time, the main active ingredient of Radix Paeoniae. Previous studies have demonstrated that PF exerts a neuroprotective effect in in vivo models of cerebral ischemia [18,19,20,21,22,23] and in in vitro models induced by H_2_O_2_ [24], MPP^+^ [25], glutamate [26], Aβ_25–35_ [27] and lipopolysaccharide [28]. They have been attributed to multiple modulation pathways, such as anti-oxidative stress, anti-inflammation, and other effects. Moreover, our recent research suggested that PF exhibited a stable and potent neuroprotective effect on cerebral ischemia injury and protected against NMDA-induced cell apoptosis and neuronal loss [23].

Thus, as a continuation, the aim of this paper was to demonstrate the neuroprotective effect of PF in vivo and in vitro, and to elucidate whether the Ca^2+^/CaMKII/CREB signaling pathway is involved in the above neuroprotective effect.

## 2. Results

### 2.1. Effect of PF on Neurological Deficit Scores and the Infarct Volume of Rats

**Figure 1:**
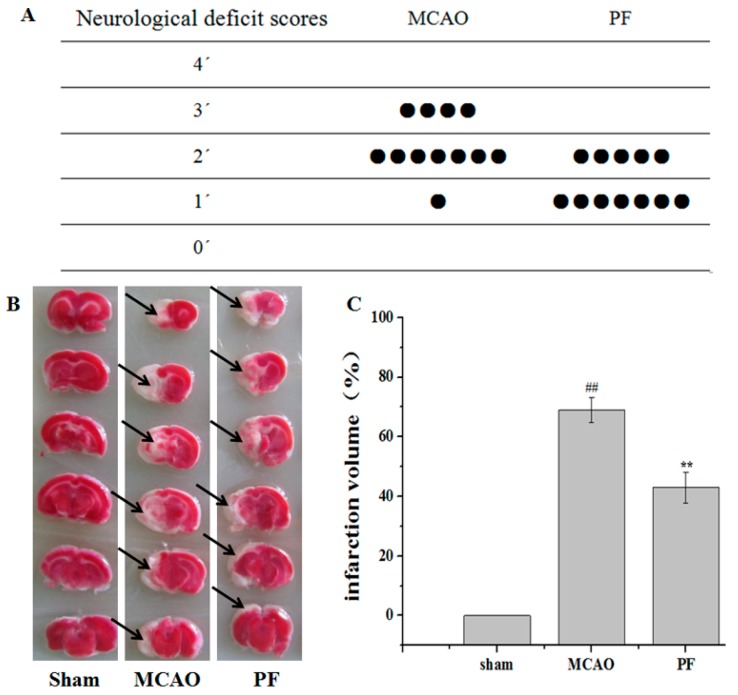
Effects of paeoniflorin (PF) on neurological deficit scores and cerebral infarct. (**A**) The neurological score of middle cerebral artery occlusion (MCAO) groups and PF groups (*n* = 12); (**B**) Representative photographs showing the cerebral infarct of rat brain slices measured by 2,3,5-triphenyltetrazolium chloride (TTC) staining. Black arrow indicated ‘‘infarcted area’’; (**C**) The infarct volume of MCAO groups and PF groups. All data were expressed as mean ± SD. ^##^ *p* < 0.01 vs. sham, ** *p* < 0.01 vs. MCAO.

The neurological deficit score and the infarct volume of rats were determined after PF treatment for 7 days. As shown in Figure 1A, rats in the middle cerebral artery occlusion (MCAO) group showed neurological deficits such as rotating while crawling and falling to the contralateral side, unable to walk without help. Administration of PF obviously improved the neurological symptoms.

**Figure 2:**
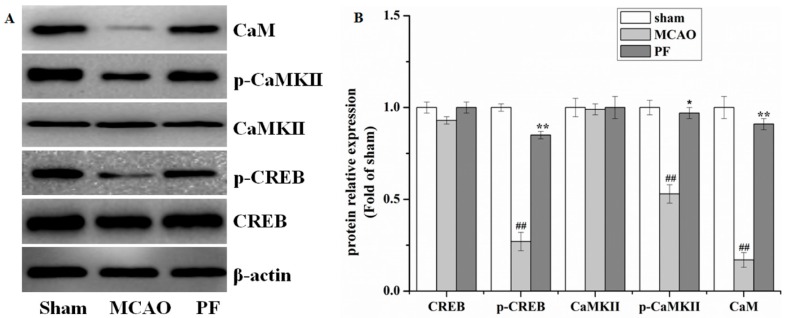
Effects of PF on the expression in the Ca^2+^/Ca^2+^/calmodulin-dependent protein kinase II (CaMKII)/cAMP response element-binding (CREB) signaling pathway in ischemic penumbra after MCAO. (**A**) Western blot and (**B**) the relative optical densities analysis of the level of CaM, CaMKII, p-CaMKII, CREB, p-CREB. β-actin were used as the internal controls. All data were presented as mean ± SD. ^##^ *p* < 0.01 vs. sham, * *p* < 0.05 and ** *p* < 0.01 vs. MCAO.

**Figure 3:**
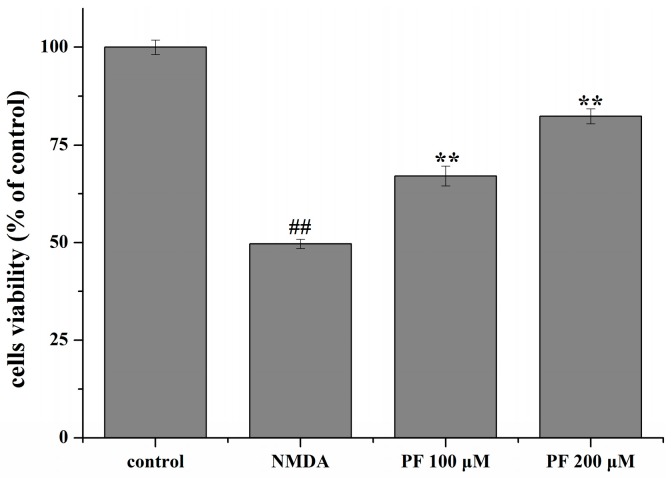
Effects of PF on *N*-methyl-d-aspartic acid (NMDA)-induced excitotoxicity in neurons as assessed by 3-(4,5-dimethyl-2-thiazolyl)-2,5-diphenyl-2-H-tetrazolium bromide (MTT). NMDA stimulation decreased cell viability in neurons. PF promoted cell survival. ^##^ *p* < 0.01 vs. control; ** *p* < 0.01 vs. NMDA. Data are mean ± SD (*n* = 8).

As shown in Figure 1B,C, 2,3,5-triphenyltetrazolium chloride (TTC) staining results revealed that the mean infarct volume in the MCAO group was significantly higher than that of the sham-operated group. Treatment with PF for 7 days significantly reduced the magnitude of ischemic lesion as compared with that of the MCAO group.

### 2.2. Effect of PF on the Expression of Proteins in the Ca^2+^/CaMKII/CREB Signaling Pathway in Ischemic Penumbra after MCAO

Western blot analysis (Figure 2A,B) revealed a decreased expression of p-CREB, p-CaMKII and CaM in the MCAO group in comparison with that of sham-operated rats, while CREB and CaMKII were unaffected. However, these changes in pathway were reversed by PF treatment.

### 2.3. Effect of PF on Cell Viability in Primary Hippocampal Neurons

Cell viability was assessed by the 3-(4,5-dimethyl-2-thiazolyl)-2,5-diphenyl-2-H-tetrazolium bromide (MTT) assay. After primary hippocampal neurons were exposed to NMDA (200 μM) for 6 h, cell viability was significantly decreased (*p* < 0.01). By contrast, incubation of cells with different concentrations of PF (100 and 200 μM) alone for 24 h increased the cell viability (*p* < 0.01, Figure 3).

### 2.4. Effect of PF on Cell Apoptosis in Primary Hippocampal Neurons

**Figure 4:**
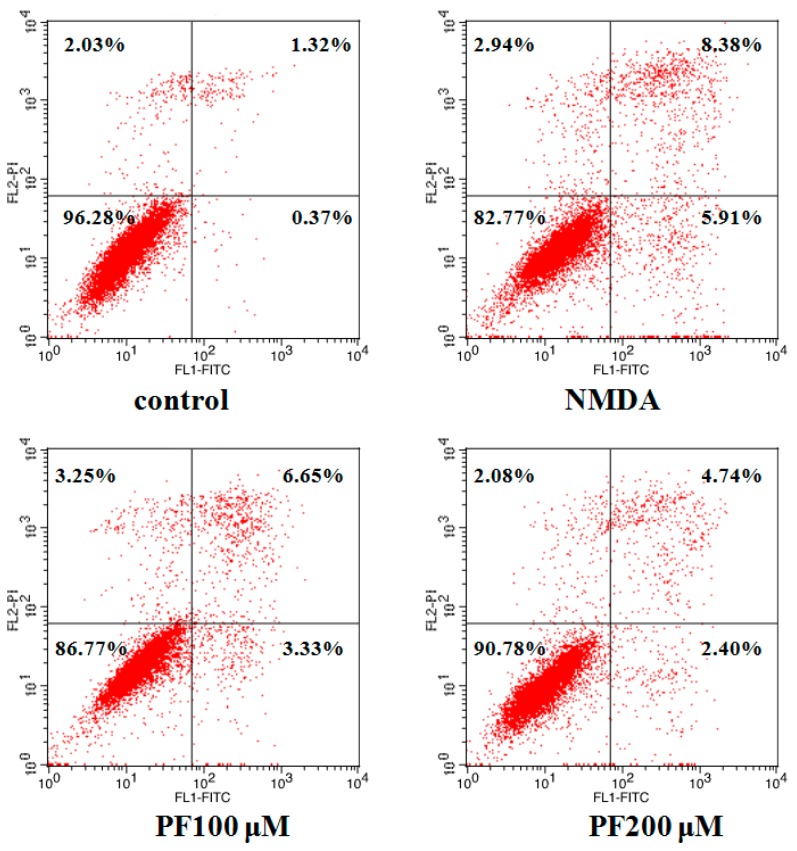
Effect of PF in NMDA-induced neurons apoptosis by annexin V/propidium iodide (PI) staining (flow cytometry analysis). NMDA stimulation increased cell apoptosis in neurons. PF promoted cell survival.

To quantitatively demonstrate the effect of PF in NMDA-induced apoptosis, annexin V/propidium iodide (PI) staining was evaluated by flow cytometric analysis. As demonstrated in Figure 4, the 3.72% of total cells was apoptosis in the control group. However, the apoptosis rate was obviously increased to 17.23% vs. the control group after incubation with NMDA. Furthermore, treatment with PF (100 and 200 μM) markedly reduced the apoptosis ratio of cells (the cell apoptosis rate was 13.23% and 9.22%, respectively).

### 2.5. Effect of PF on Intracellular Ca^2+^ Concentration

**Figure 5:**
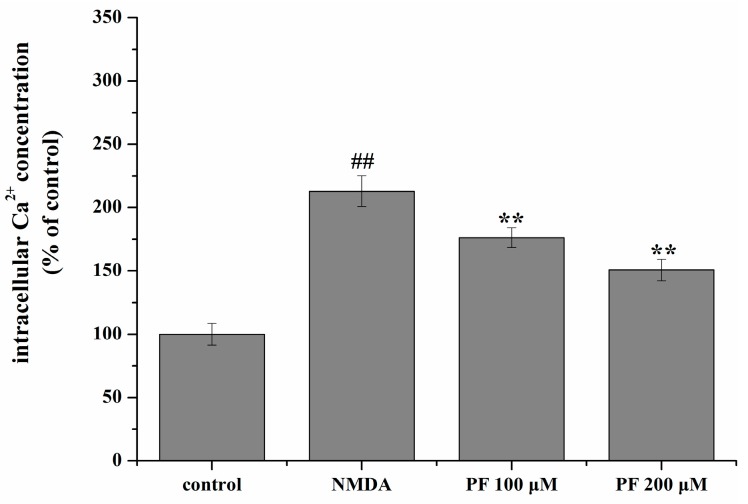
Effect of PF on intracellular Ca^2+^ concentration in NMDA-induced PC12 cells. Intracellular Ca^2+^ concentration was measured by the Fura-2/AM fluorescent technique. All data were presented as mean ± SD (*n* = 6). ^##^ *p* < 0.01 vs. control; and ** *p* < 0.01 vs. NMDA.

As shown in Figure 5, the concentration of intracellular Ca^2+^ increased significantly after NMDA treatment compared with the control group (213% of the control value, *p* < 0.01). PF treatment significantly decreased the intracellular Ca^2+^ concentration (176% and 151% of the control value, respectively).

### 2.6. Effect of PF on the Expression of Proteins in the Ca^2+^/CaMKII/CREB Signaling Pathway in Primary Hippocampal Neurons after NMDA-Induced Excitotoxicity

**Figure 6:**
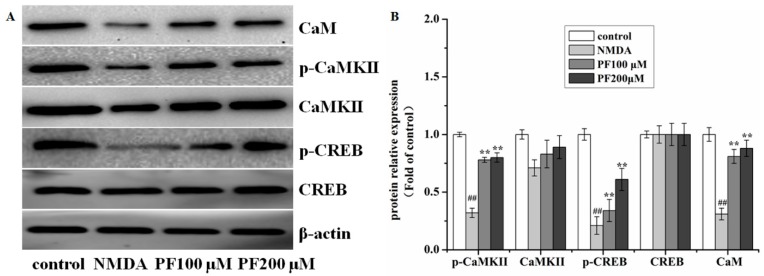
Effects of PF on the expression in the Ca^2+^/CaMKII/CREB signaling pathway in neurons after NMDA-induced excitotoxicity. (**A**) Western blot and (**B**) the relative optical densities analysis of the level of CaM, CaMKII, p-CaMKII, CREB, p-CREB. β-actin was used as the internal controls. All data were presented as mean ± SD. ^##^ *p* < 0.01 vs. control;, ** p < 0.01 vs. NMDA.

To further assess whether PF pretreatment could modulate the Ca^2+^/CaMKII/CREB signaling pathway in vitro, we also examined the expression of proteins in primary hippocampal neurons after NMDA-induced excitotoxicity. Consistent with results in vivo, pretreatment of PF changed the levels of CaM, CaMKII, p-CaMKII, CREB and p-CREB (Figure 6).

## 3. Discussion

Cerebral ischemic injury is a complicated cascade process, which is mainly differentiated into two processes: the initial tissue injury caused by ischemia and the secondary tissue injury inflicted by ischemia reperfusion [29]. The secondary tissue injury aggravates cerebral ischemic injury and compelling evidence indicates a high intracellular Ca^2+^ influx in this process. Ca^2+^ influx leads to the elevation of the Ca^2+^ concentration in neurons which disrupts the balance of ionics, then activates various Ca^2+^-dependent enzymes (such as CaMKII, nitric oxide synthase, calcineurin) and Ca^2+^-binding proteins. Ca^2+^ overload into neurons induced by ischemic insult may excessively activate Ca^2+^/CaM-dependent pathways and lead to irreversible cell damage [6]. Thus, therapeutic strategies targeting the secondary tissue injury and intracellular Ca^2+^ concentration could inhibit the progression of brain injury, providing an extended therapeutic strategy for neuroprotection.

Neurons are the core components of the central nervous system and can form a neural network through dendrites and axons. Neurons, along with the neural network, are the basis for neurological functions [30]. In addition, neurons are sensitive to ischemia and hypoxia, thus they are used in the in vitro neural system to conduct related research.

NMDA is an excitatory neurotransmitter, which acts on *N*-methyl-d-aspartate receptors (NMDAR) of postsynaptic neurons, activates the calcium channel controlled by the receptor, and results in calcium overload. Under pathological conditions, Ca^2+^ internal flow leads to acute edema in the cell, and secondary cell toxicity ultimately triggers neuron apoptosis. It has been proved that NMDA can result in rising Ca^2+^ concentration in the cytoplasm and marked elevation in Ca^2+^ then causes neuronal death [31,32,33]. Thus, NMDA is often used as an excitotoxicity-inducing agent to study the molecular mechanism and to develop drugs for ischemic stroke therapy.

In addition, CaM is activated by combining it with Ca^2+^ followed by intracellular Ca^2+^ overload, and the kinase is activated by autophosphorylation at Thr286; then the CREB protein is activated. CREB is a transcription factor present in numerous tissues, which plays a regulatory role in neuronal survival, precursor proliferation, neurite outgrowth, and neuronal differentiation in neurons [14,15,16].

Thus, protecting neurons may improve neurological functions which is beneficial to neurodegeneration diseases. In a previous study, paeoniflorin protects against ischemia-induced neurons apoptosis, mainly via regulating the Bcl-2/Bax signal pathway [34], the Ras/MEK/ERK signaling pathway [18] and the NF-κB-Mediated signal pathway [21]. In addition, Wang et al. [35] and Mao et al. [31] demonstrated that paeoniflorin could suppress intracellular Ca^2+^. Therefore, we hypothesized that PF may protect against cerebral ischemia via regulating the expression of the Ca^2+^/CaMKII/CREB signaling pathway. Thus, in the present study, we used MCAO and NMDA-induced excitotoxicity models of ischemic stroke to further investigate the neuroprotective effects of PF as well as the underlying mechanism, by focusing on the Ca^2+^/CaMKII/CREB signaling pathway, that was closely related to anti-apoptotic signaling. In an in vitro study, we investigated the neuroprotective effects of PF against NMDA-induced neurotoxicity in neurons and verified its neuroprotective effects. Previous studies reported that PF exerts a neuroprotective effect in in vitro models induced by H_2_O_2_ [24], MPP^+^ [25], glutamate [26], Aβ_25–35_ [27] and lipopolysaccharide [28]. In line with these studies, our results suggest that the anti-NMDA induced excitatory toxic injury may be involved in the neuroprotective effect of PF. However, these inhibitions were not in a crucial concentration-dependent manner.

Furthermore, in accordance with the hypothesis, in this study, we observed that p-CaMKII, and p-CREB were markedly decreased and these reductions were attenuated by PF treatment in both the MCAO and NMDA-induced excitotoxicity models, but the expression of CaMKII and CREB was not notably changed, suggesting that they exerted their function through the phosphorylation rather than regulation of their protein expression. It was found that PF also regulated downstream proteins along the Ca^2+^/CaMKII/CREB signaling pathway, including Bax, BcL-2, Bad and caspase 3 [23]. In conclusion, we speculated that PF attenuates neuronal apoptosis, which phosphorylates CaMKII and CREB protein, and finally inhibits the activation of downstream target molecules or inhibited cell apoptosis, including Bax, BcL-2, Bad and caspase 3.

## 4. Materials and Methods

### 4.1. Reagents and Animals

**Figure 7:**
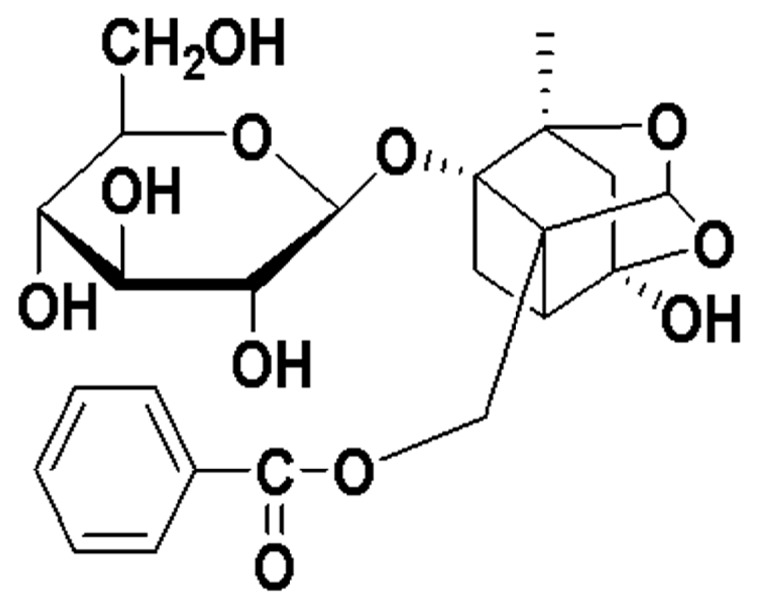
Chemical constitution of paeoniflorin (PF).

PF (>98% purity, Figure 7) was purchased from the National Institute for the Control of Pharmaceutical and Biological Products (Beijing, China). Fluo-2/AM, dimethyl sulfoxide (DMSO), 3-(4,5-dimethylthiazol-2-yl)-2,5-di-phenyl (MTT) and NMDA were purchased from Sigma-Aldrich (St. Louis, MO, USA). Trypsin, fetal bovine serum and penicillin-streptomycin were purchased from hyclone (Logan, UT, USA). The annexin V/propidium iodide (PI) apoptosis assay kit was obtained from Roche Diagnostics (Indianapolis, IN, USA). Neurobasal^®^ medium, B-27^®^ serum-free supplement and GlutaMAX™-I were bought from Life Technologies (Foster, CA, USA). Antibodies to CREB, p-CREB, CaMKII, p-CaMKII, CaM and β-actin were bought from Cell Signaling Technology (Danvers, MA, USA). Horseradish peroxidase (HRP)-conjugated goat anti-rabbit (mouse) IgG were from Xiamen Lulong Biotech Co., Ltd. (Xiamen, China). Polyvinylidene fluoride membrane was from Merck KGaA (Darmstadt, Germany). All other reagents were from Beyotime Institute of Biotechnology (Nanjing, China) unless otherwise stated.

A total of twenty-four male Sprague-Dawley rats weighing 260 ± 20 g were bought from the Laboratory Animal Center of Fujian University of Traditional Chinese Medicine (Fuzhou, China). All animals were housed in the standard laboratory animal conditions (humidity: 55% ± 5%, room temperature: 22 ± 2 °C with a 12:12 h light–dark cycle). Furthermore, all experimental protocols and handling procedures of animals were approved by the Ethics Committee of Fujian University of Traditional Chinese Medicine (Fuzhou, China).

### 4.2. Middle Cerebral Artery Occlusion and Reperfusion (MCAO) Model

Rats were fasted 12 h before surgery but were allowed free access to water. All animals were anesthetized with 10% chloral hydrate solution (0.3 mL/100 g, i.p.). Then the MCAO model was performed for the induction of focal cerebral ischemia as previously described [15,36,37,38]. Briefly, a 3-0 silicon rubber-coated nylon monofilament (Guangzhou Jialing Biotechnology Co., Ltd., Guangzhou, China) was inserted into the internal carotid artery to occlude the origin of the left middle cerebral artery until light resistance was felt (18–20 mm from common carotid artery bifurcation). After 2 h of MCAO, the monofilament was removed to implement reperfusion. Rats of the sham-operated group underwent the same surgical operation except that the suture was not inserted.

### 4.3. Experimental Groups and Treatment

Rats that underwent the MCAO were divided randomly into three groups: the MCAO group, the PF group and the sham-operated group. All rats were administrated twice per day for 7 days as follows: rats in the PF group were intraperitoneal injected PF 5 mg/kg; rats in the sham-operated group and MCAO group received the same volume of normal saline.

### 4.4. Evaluation of Neurological Deficit

Neurobehavioral deficits of rats were evaluated on a five-point scale as described previously [23,36,37,38]. The scale was as follows: 0, no neurological symptoms; 1, unable to completely extend the front jaw on the other side; 2, rotating while crawling and falling to the contralateral side; 3, unable to walk without help; and 4, unconsciousness. In order to exclude the interference of operative failures, rats with a score of 1–3 points were included in this study.

### 4.5. Measurement of Ischemic Infarct Area

After neurological evaluation, rats were deeply anesthetized and then were decapitated to take out the brain quickly. Then it was done as described previously [36,37,38]. Briefly, the brains were cut into 2 mm slices and incubated with 0.2% TTC (T8877; Sigma-Aldrich, St. Louis, MO, USA) in phosphate-buffered saline (PBS, PH 7.4) at 37 °C for 1 h. The image of each slice was captured and calculated by Motic Med 6.0 Digital Medical Image Analysis system (Motic Instruments Inc., Richmond, BC, Canada).

### 4.6. Primary Hippocampal Neuron Culture and NMDA-Induced Excitotoxicity Model

Primary cultures of neurons were obtained from the cerebral hippocampi of newborn SD rats within 24 h. The procedures were the same as described previously [23,38]. In brief, hippocampi tissues were isolated and digested in 2 mg/ml of papain (Sigma). The obtained cell suspension was plated onto a poly-d-lysine (Sigma) coated 96-well plate or 6-well plate and cultivated in neurobasal^®^ medium (Life Technologies) supplemented with 2% B-27^®^ serum-free supplement (Life technologies) and 0.5 mM GlutaMAX™-I (Life Technologies) at 37 °C in a humidified atmosphere of 5% CO_2_. Half of the culture medium was replaced every third day.

The NMDA-induced excitotoxicity model was performed as in our previous research [38]. After 7 days in culture, the original culture medium was collected and replaced by NMDA solution for 20 min. Then neurons were washed with PBS and returned to the original culture medium with PF (100 and 200 μM) for another 24 h.

### 4.7. Cell Viability Assay

The cell viability was assessed by MTT assay. Briefly, after treatment, 10 μL MTT solution (5 mg/mL) was added into each well for an additional 4 h incubation. Then MTT reagent was replaced with DMSO (100 μL per well) carefully to dissolve formazan crystals. Absorbance at 570 nm was measured in a microplate reader (Infinite M200 Pro, Tecan, Männedorf, Switzerland). Results were expressed as the percentage of the absorbance of control cells, which was considered as 100%.

### 4.8. Flow Cytometric Analysis

After treatment, cells were collected and quantitated according to the manufacturer’s protocol. Briefly, cells were resuspended in binding buffer and stained with annexin V/PI for 15 min. Then samples were analyzed by a flow cytometer with an excitation wavelength of 488 nm and an emission wavelength of 530 nm (Becton-Dickinson, Bedford, MA, USA). Apoptotic cells were expressed as a percentage of the total number of cells.

### 4.9. Intracellular Ca^2+^ Measurement

After treatment, the cells were washed with D-PBS and incubated with the complete medium containing 5 μm Fura-2/AM at 37 °C for 45 min. Subsequently, the cells were washed with D-PBS containing 0.2% bovine serum albumin (BSA). Then, the cells were incubated at 37 °C for another 5 min prior to measurement. Intracellular Ca^2+^ concentration was determined by alternating excitation wavelengths of between 340 and 380 nm with emission at 510 nm in a fluorescence spectrophotometer (Infinite M200 Pro). Intracellular Ca^2+^ concentration was expressed as a percentage of non-treated control.

### 4.10. Western Blot Analysis

After treatment, cells and cortex tissues of ischemic brain were harvested and lysed by radio-immunoprecipitation assay (RIPA) lysis buffer containing a protease inhibitor cocktail (PMSF) on ice; they were then centrifuged at 12,000× *g* for 15 min at 4 °C. Then, equal amounts of proteins were electrophoresed on 12% density sodium dodecyl sulfate, sodium salt (SDS) acrylamide gels, transferred to a polyvinylidene fluoride (PVDF) membranes. The membranes were blocked with 5% skim milk (Cell Signaling Technology, Danvers, MA, USA) in tris-buffered saline and tween 20 (TBST) for 2 h, followed by overnight incubation with antibodies to CREB (1:1000), p-CREB (1:1000), CaMKII (1:1000), p-CaMKII (1:1000), CaM (1:500) and β-actin (1:1000). Membranes were then incubated for 2 h at room temperature with HRP-conjugated secondary antibody (Proteintech Group, Chicago, IL, USA, 1:7000). Finally, all specific bands were visualized using the electrochemical luminescence (ECL) western detection reagents on Image Lab analysis software (Bio-Rad, Philadelphia, PA, USA). β-actin was used as a loading control. Three repeats of the experiments were performed.

### 4.11. Statistical Analysis

Data were presented as the mean ± standard deviation (SD). One-way analysis of variance (ANOVA) (SPSS 20.0 statistical software, IBM, Chicago, IL, USA) followed by a post hoc Fisher’s least significant difference (LSD) test to evaluate multiple group difference. *p* < 0.05 was considered to indicate statistical significance.

## 5. Conclusions

In summary, the results of this study demonstrated that the neuroprotective effects of PF on cerebral ischemia-reperfusion injury were associated with the improvement of CaMKII and CREB activation, at least in part. The findings may represent a novel mechanism of PF in focal cerebral ischemia-reperfusion injury in rats. Whether other pathways or mechanisms participated in the beneficial effects of PF on ischemic brain injury needs further investigation.
